# 2D SnO_2_ Nanosheets: Synthesis, Characterization, Structures, and Excellent Sensing Performance to Ethylene Glycol

**DOI:** 10.3390/nano8020112

**Published:** 2018-02-16

**Authors:** Wenjin Wan, Yuehua Li, Xingping Ren, Yinping Zhao, Fan Gao, Heyun Zhao

**Affiliations:** 1 College of Materials Science and Engineering, Yunnan University, Kunming 650091, China; wenjwan@163.com (W.W.); skyzyp@foxmail.com (Y.Z.); f_gao_yn@sohu.com (F.G.); 2 Advanced Measurement and Analysis Center of Dali University, Dali 671200, China; yuehua_li99@sohu.com; 3 Yunnan Security and Technology Co., Ltd., Kunming 650033, China; 4 Yunnan Key Laboratory for Micro/Nano Materials and Technology, Yunnan University, Kunming 650091, China

**Keywords:** tin dioxide, nanosheets, hydrothermal route, gas sensor, ethylene glycol

## Abstract

Two dimensional (2D)SnO_2_ nanosheets were synthesized by a substrate-free hydrothermal route using sodium stannate and sodium hydroxide in a mixed solvent of absolute ethanol and deionized water at a lower temperature of 130 °C. The characterization results of the morphology, microstructure, and surface properties of the as-prepared products demonstrated that SnO_2_ nanosheets with a tetragonal rutile structure, were composed of oriented SnO_2_ nanoparticles with a diameter of 6–12 nm. The X-ray diffraction (XRD) and high-resolution transmission electron microscope (FETEM) results demonstrated that the dominant exposed surface of the SnO_2_ nanoparticles was (101), but not (110). The growth and formation was supposed to follow the oriented attachment mechanism. The SnO_2_ nanosheets exhibited an excellent sensing response toward ethylene glycol at a lower optimal operating voltage of 3.4 V. The response to 400 ppm ethylene glycol reaches 395 at 3.4 V. Even under the low concentration of 5, 10, and 20 ppm, the sensor exhibited a high response of 6.9, 7.8, and 12.0 to ethylene glycol, respectively. The response of the SnO_2_ nanosheets exhibited a linear dependence on the ethylene glycol concentration from 5 to 1000 ppm. The excellent sensing performance was attributed to the present SnO_2_ nanoparticles with small size close to the Debye length, the larger specific surface, the high-energy exposed facets of the (101) surface, and the synergistic effects of the SnO_2_ nanoparticles of the nanosheets.

## 1. Introduction

In recent years, novel nanostructures and their applications of nanomaterials have attracted a great deal of research activity because they have created unexpected functionality in terms of physicochemical properties. The main interest focus has been on the rational design and precise control over specialized morphologies of nanomaterials with tailored properties. Among various nanostructures, dimensionality (D) of the nanostructures is a key factor, which significantly controls the physicochemical properties of the nanomaterials’ behavior. Until now, zero-dimensional (0D) (quantum dots), one-dimensional (1D) (nanorods, nanobelts, nanowires), and three-dimensional (3D) crystals are abundantly reported. In comparison with other nanostructured materials, the applications of 2D nanostructured materials as building blocks make possible the design and synthesis of novel nanostructured materials with versatile and tailorable physicochemical properties, such as sensing performance [1,2].

Tin dioxide (SnO_2_), due to its intrinsic nonstoichiometry from oxygen vacancies, is regarded as an n-type metal oxide semiconductor with a wide direct band gap of 3.6 eV at 300 K, and has grabbed more attention for remarkable physical and chemical properties, including high chemical stability, mechanical properties, and good optical and electrical properties [3,4]. In the past few years, SnO_2_ have been widely used in gas sensors [5], supercapacitors [6], solar cells [7], humidity sensors [8], lithium ion batteries [9], and so on. As a classic gas-sensing material, SnO_2_ has been widely used in gas sensors due its lower cost, higher chemical sensitivity, faster gas response, and good stability. Generally, SnO_2_ displays excellent gas sensing properties to volatile organic compound vapors and toxic gases, such as isopropanol [5], ethanol [10], ammonia [11], formaldehyde [12], and so on. However, there are still some problems that need to be solved for SnO_2_ gas sensors, such as the poor selectivity, slow response or recovery speed, and lower response. As is well known, many factors, such as grain size, microstructures, morphologies, and so on, all have a great influence on the gas-sensing performance of SnO_2_ sensors [13]. Recently, SnO_2_, with various nanostructures and morphologies, including nanoparticles [14], nanosheets [1], nanowires [15], nanorods [16], nanofibers [10], nanospheres [17], and nanofilms [18], have been developed to investigate the effects of the structure and morphology on the gas-sensing performance. Among them, nanosheets have aroused people’s interest because of the large and continuous surface which provides a higher specific surface and contains an abundance of active sites which are a benefit to physical and chemical adsorption. Secondly, those small-sized SnO_2_ nanoparticles that are organized in the SnO_2_ nanosheets, usually lead to a high specific surface and novel physical and chemical effects [19]. Meanwhile, high-energy exposed facets of SnO_2_ crystals can also promote the reaction of gas molecules on the surface. Taking those into consideration, SnO_2_ nanosheets assembled by nanoparticles can be expected to lead to excellent sensing performance to volatile organic compound vapors. Recently, it was reported that many physical and chemical methods have been employed to synthesize different SnO_2_ nanosheets. Zeng et al. synthesized hierarchical SnO_2_ hollow nanosheets by a simple anneal-etching method and the as-synthesized products showed improved gas-sensing properties to acetone at the optimum working temperature of 300 °C [2]. Dong et al. reported that porous Pt-functionalized SnO_2_ sheets were synthesized by a combustion method, and it exhibited a significant enhancement for isopropanol gas detection [20]. Li et al. prepared SnO_2_ nanosheets with a polypyrrole coating via an in situ growth of electro-spun nanofibers which contained SnCl_2_ on the substrates under hydrothermal conditions. The as-prepared products displayed good gas-sensing performance to ammonia [11]. However, those synthesis methods usually need substrates, surfactants, and a combustion improver, which are harmful to the health of human beings and the environment. Additionally, the complex operation and expensive equipment are also drawbacks of those synthesis methods. Therefore, it is necessary to explore simple, low-cost, and pollution-free preparation methods of SnO_2_ nanosheets.

Although, as one of the most important organic solvents, ethylene glycol is widely used as the raw materials of antifreeze, coolants, and the solvent of dyes and inks, the vapor is toxic and can cause serious damage to human health. The hydroxyl group contained in ethylene glycol will be oxidized into glycolic acid and then into oxalic acid with their working temperature of 80–90 °C. It will induce respiratory difficulty, pulmonary edema, and so on. It also negatively affects the nervous system and even causes death. Thus, it is necessary to develop various glycol vapor detection techniques and devices. However, to the best of our knowledge, gas sensors for glycol detection are rarely reported.

In the present work, we demonstrated a one-step hydrothermal method without substrates or surfactants for SnO_2_ nanosheet synthesis at a lower temperature of 130 °C. The morphology, crystal phase, microstructure, and spectral characteristics of the as-prepared SnO_2_ nanosheets were investigated. It is found that the dominant exposed surface of the SnO_2_ nanoparticles is (101), but not (110). The gas-sensing experiments of the SnO_2_ nanosheets towards ethylene glycol were carried out to envisage the sensing performances, including sensitivity, response and recovery times, and selectivity, which explore the potential applications for a high-performance sensor to detect glycol in environmental gas monitoring. A possible mechanism of the excellent gas-sensing performance for the SnO_2_ nanosheets was also discussed in detail.

## 2. Materials and Methods

### 2.1. Chemical Reagent

All chemical reagents used were of analytical grade and without further purification. Sodium stannate was purchased from Sinopharm Chemical Reagent Co., Ltd. (Shanghai, China). Sodium hydroxide was purchased from Guangzhou Jinhuada Chemical Reagent Co., Ltd. (Guangzhou China). Absolute ethanol was purchased from Tianjin Fengchuan Chemical Reagent Technologies Co., Ltd. (Tianjin, China).

### 2.2. Syntheses Process of 2D SnO_2_ Nanosheets

The *2D* SnO_2_ nanosheets were synthesized via a facile hydrothermal process with neither substrates nor surfactants present. In the typical synthesis process, 20 mL of uniform sodium stannate (Na_2_SnO_3_·4H_2_O) suspension solution (0.376 mol/L) and 20 mL of transparent sodium hydroxide (NaOH) solution (0.7 mol/L) were separately prepared. Then, the sodium hydroxide solution was added into the sodium stannate solution with stirring for 20 min. After that, 40 mL absolute ethanol was added into the mixed solution slowly and stirred for 30 min to a form a white precursor solution with a pH value above 13 measured by an extensive pH indicator paper. The precursor solution was transferred into a 100 mL Teflon-lined autoclave and the temperature was kept at the 130 °C for 48 h. The as-prepared product was cooled to room temperature naturally. Subsequently, the resulting samples’ collected precipitate was washed several times with deionized water and absolute ethanol. Finally, the as-prepared product was dried at 80 °C for 24 h for further characterization.

### 2.3. Characterization of 2D SnO_2_ Nanosheets

The phase of the as-prepared SnO_2_ nanosheets was characterized by the X-ray diffraction pattern (XRD) using a Rigaku D/MAX-RA diffractometer (Rigaku Corporation, Tokyo, Japan) with copper target Kα radiation (*λ* = 1.54056 Å) at 40 kV and 100 mA. The products were scanned from 10° to 90° at a scan rate of 0.02 °/s. The surface morphologies were performed by an FEI Quanta 200 field emission scanning electron microscope (FESEM, Hillsboro, OR, USA). The detail structure of the nanosheets was carried out on transmission electron microscopy (TEM), high-resolution transmission electron microscopy (HRTEM), and selected-area electron diffraction (SAED) by a JEM-2100UHR (Akita City, Tokyo, Japan) at an accelerating voltage of 200 kV. The spectral characteristics of products were tested by Fourier transform infrared spectra (FT-IR) within the wavenumber range from 4000 to 400 cm^−1^ by FTS-40 Fourier transform infrared spectrometer (Digilab/BioRad, Cambridge, MA, USA) and the Raman scattering spectra was obtained by a Renishaw INVIA Laser Micro-Raman spectrometer (Gloucestershire, UK). Ultraviolet-visible spectroscopy (UV-VIS) was measured with a UV-2401PC spectrophotometer (Shimadzu, Japan). X-ray photoelectron spectroscopy (XPS) was performed at room temperature using a PHl X-tool (Ulvac-Phi, Tokyo, Japan) which takes an Al Kα X-ray beam as the excitation source under 250 W of power. The Brunauer-Emmett-Teller nitrogen adsorption isotherm (BET) was obtained via a Micromeritics Gemini VII (Surface Area and Porosity System, Atlanta, GA, USA) apparatus at 300 °C. The specific area and the distribution of the pore sizes were analyzed by the Barett-Joyner-Halenda method (BJH).

### 2.4. Sensor Fabrication and Measurement of Gas-Sensing Performance

**Figure 1:**
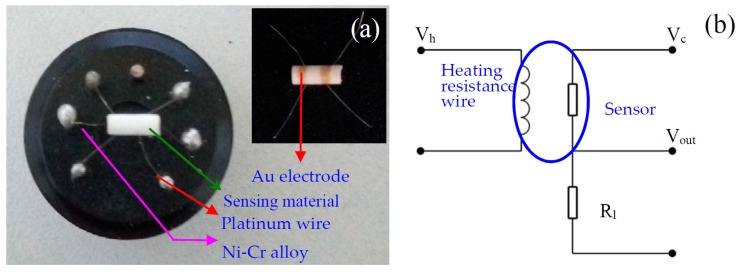
(**a**) Images of the sensor. Inset: alumina tube with two Au electrodes and four Pt wires installed; (**b**) The circuit diagram of the gas-sensing measurement system.

The indirect-heating structural gas sensor (shown in Figure 1a) was elected to investigate the response to various VOCs. The gas sensor was fabricated as follows: an appropriate volume of absolute ethanol and deionized water was firstly added into the as-prepared products to form a uniform white slurry. Then, the slurry of products was painted onto the surface of an alumina tube with two Au electrodes and four Pt wires installed. The alumina tube is 4 mm in length, 1.2 mm in external diameter, and 0.8 mm in internal diameter. The thickness of the sensitive body is 0.5 mm. After that, the preliminary sensor was roasted at a temperature of 400 °C for 2 h. Subsequently, a Ni-Cr alloy coil, which acts as a heater to adjust the operating temperature from room temperature to 600 °C, controlled by tuning the heating voltage, crossed the alumina tube. Before measuring the gas sensing properties, the sensor was aged at 500 °C for seven days in the air. 

The gas-sensing properties were tested in a chamber through which a controlled atmosphere was allowed to flow. The electrical response of the sensor was measured with an automatic test system (JF02F, Jin Feng Electronics of Sino-Platinum Metals Company Limited, Yunnan, China). The export signals of the sensor was measured by using a conventional circuit (as shown in Figure 1b) in which the element was connected with an external resistor in series at a circuit voltage (V_c_). According to Figure 1b, the electrical resistance of the sensor can be obtained. The gas response *S* is defined as the ratio of the electrical resistance in air (*R_a_*) to that in indoor targeted gases (*R_g_*):

*S* = *R_a_*/*R_g_* (1)

Additionally, the gas-sensing properties test was carried out under a relative humidity between 40% and 60%.

## 3. Results and Discussion

### 3.1. Morphology, Structure, and Formation Mechanism of SnO_2_ Nanosheets

**Figure 2:**
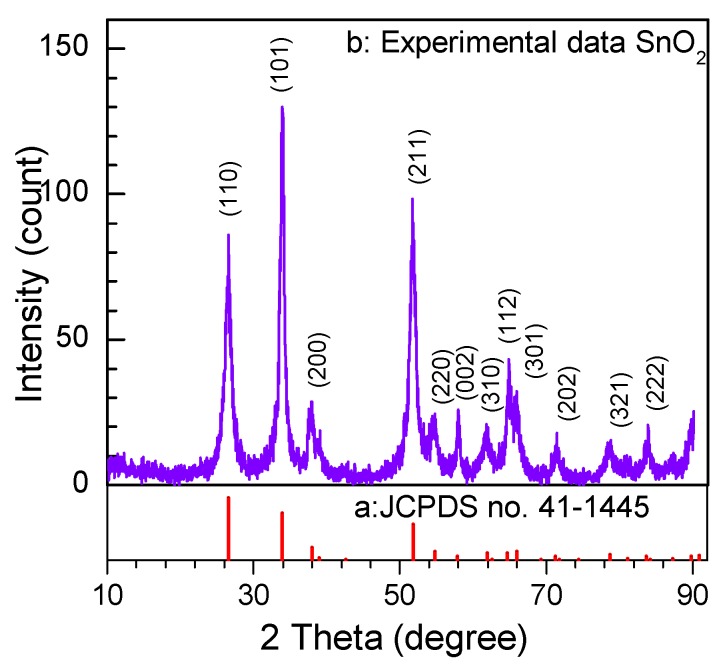
XRD pattern of the as-prepared SnO_2_ nanosheets.

The XRD of the as-prepared product is shown in Figure 2. It exhibits a texture effect corresponding to anisotropy and orientation of the crystal shape. All diffraction peaks are indexed to the phase of rutile-type SnO_2_ with the tetragonal lattice parameters of *a* = *b* = 4.738 Å, c = 3.187 Å and the space group of P_42/mnm_ (136) according to JCPDS file no. 41-1445. No peaks of other impurities exhibit the high purity of the as-prepared products. The relative high intensity of peaks indicates a good crystallinity of the as-prepared SnO_2_ nanosheets, and a large width of the full width at half maximum (FWHM) of the peaks suggests the small grain size of the SnO_2_ nanosheets, which can calculate via Scherer’s equation:

*D* = *kλ*/*βcosθ* (2)

where *k* is the Scherer constant taking the value of 0.89, *λ* is the wavelength of the X-ray source taking the value of 1.5406 Å, *β* is the full width at half maximum (FWHM), and *θ* is the Bragg angle. According to Scherer’s equation, the mean particle size of the as-prepared products that correspond to the (101) plane diffraction peak is about 7.8 nm. The small size effect of the nanoparticles may lead to a large specific surface which is beneficial to the performance of the gas sensor. On the other hand, it is obvious from Figure 2 that the (101) peak is relatively stronger than (110), which demonstrates that the dominant expose surface is (101), but not (110). It is an important result that the exposed special high-energy surface of SnO_2_ will result in a good effect on the gas-sensing performance.

**Figure 3:**
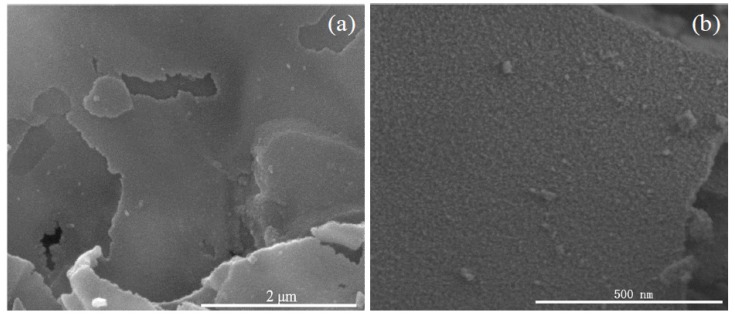

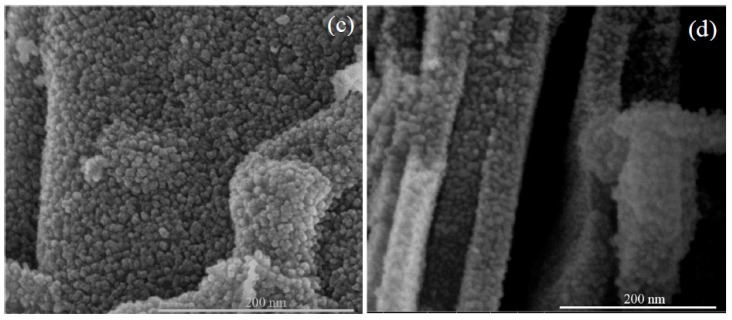
FESEM images of as-prepared SnO_2_ nanosheets. (**a**) Panoramic top-view image; (**b**) FESEM image; (**c**) High-magnification FESEM images; (**d**) Cross-sectional image.

The FESEM images of the as-prepared products are shown in Figure 3. As shown in Figure 3a, the panoramic image reveals that the as-prepared nanosheets display a large and continuous area, and a smooth surface. From Figure 3b, it is clear that there is an abundance of protuberant pots on the surface of the nanosheets, which illustrates that the nanosheets are formed by a mass of small SnO_2_ nanoparticles. In order to further observe the microstructure of the nanosheets, FESEM was employed to acquire the higher magnification image, as shown in Figure 3c. It can be seen that the nanosheets are assembled by an abundance of uniformly-sized particles with diameters of 6–12 nm. A cross-edge section FESEM image of the nanosheets, as shown in Figure 3d, displays that SnO_2_ nanoparticles are densely distributed and tightly packed to form the layers of nanosheets. Moreover, it is obvious that the SnO_2_ nanosheet is tightly combined by 4–8 nanoparticle layers with an apparent interface in the center of the nanosheet, and the edge thickness of the nanosheet is about 58.3 nm. All of these observations are in good agreement with the XRD results. Due to the large and continuous area of nanosheets and the small size of nanoparticles, the as-prepared nanosheets might yield good gas-sensing properties.

**Figure 4:**
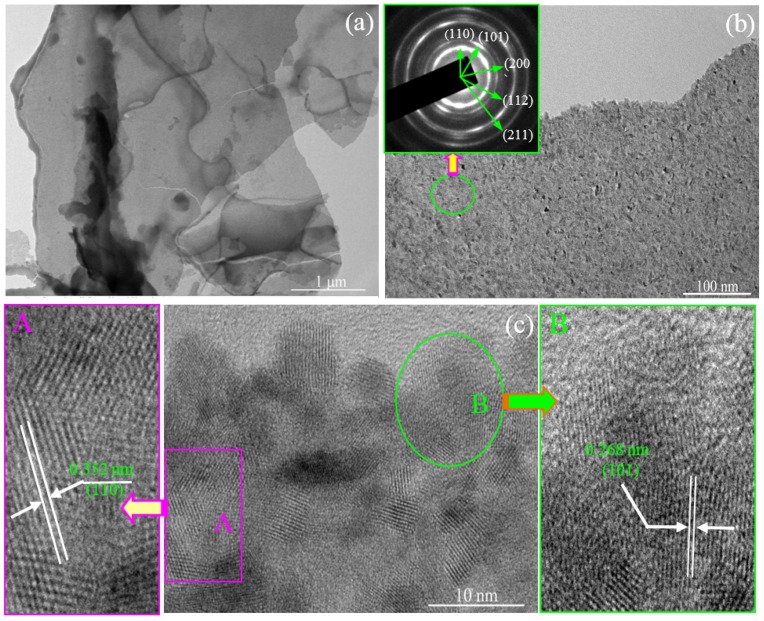
TEM images of the as-prepared SnO_2_ nanosheets. (**a**) Low-magnification TEM image; (**b**) High-magnification TEM image. Inset: the image of selected area electron diffraction; (**c**) Lattice-revolved HRTEM image. The further enlarge HRTEM image of the marked area A and B showing the interplanar spacing of SnO_2_ nanoparticles with the (110) and (101) crystal face.

Detailed morphologies and microstructures for the as-prepared products are shown in Figure 4. As the low-magnification TEM image shows in Figure 4a, the morphology of the as-prepared nanosheets depict a large and continuous area with a smooth surface. At the same time, the see-through image suggests a thin thickness of the nanosheets. The high-magnification TEM image is shown as Figure 4b, which displays the dense and wide distribution of the nanoparticles. From Figure 4b, an abundance of protuberant pots can be seen on the surface of the nanosheets. The inset image of Figure 4b shows the corresponding selected area electron diffraction (SAED) pattern of the SnO_2_ nanosheets. This suggests the polycrystalline nature of the as-prepared products, which could be assigned to (110), (101), and (211) lattice planes of tetragonal rutile-type SnO_2_. HRTEM was employed for further observation of the detailed microstructure of the as-prepared SnO_2_ nanosheets. As shown in Figure 4c, these nanoparticles are combined with each other closely. The nanoparticles exhibit approximate uniform grain size, whose diameter ranges from 6 to 10 nm. Those results are in accordance with FESEM and XRD results. Furthermore, high-quality and clear lattice fringes of single SnO_2_ nanoparticles are observed in Figure 4c. However, it is obvious that the *d*-spacing between two adjacent lattice fringes of the SnO_2_ nanoparticles is not uniform at first glance. It can be divided into two cases according to the space of the adjacent lattice fringes, which are marked as A and B areas, as shown in enlarged images, respectively. The image marked A depicts a clear and uniform lattice fringe with a *d*-spacing of 0.352 nm, corresponding to the (110) lattice planes of SnO_2_ with a tetragonal structure. This indicates that the exposed facet of the SnO_2_ nanoparticles is the (110) plane and the oriented growth is preferentially along the [001] direction [21]. Additionally, most of the other SnO_2_ nanoparticles exhibit a narrower *d*-spacing as shown in area B. The *d*-spacing is approximately 0.268 nm, which corresponds to the (101) lattice plane. These results prove that the preferential growth lattice plane is (101), rather than the conventional (110) plane. This discovery agrees nicely with the XRD results that the intensity of the (101) peak is stronger than that intensity of the (110) plane.

A possible formation process of the SnO_2_ nanosheets can be speculated. As an unstable chemical compound, after the sodium stannate is added to alkaline deionized water, the Sn^4+^ ions are rapidly hydrolyzed and oxidized to form the oversaturated solution of dissoluble Sn(OH)_6_^2−^ complex ions according to the chemical reactions from (3) to (5) [21]:

Na_2_SnO_3_ + 2H_2_O → H_2_SnO_3_↓ + 2NaOH (3)

SnO_3_^2−^ + 3H_2_O → Sn(OH)_4_↓ (4)

Sn(OH)_4_ + 2OH^−^ → Sn(OH)_6_^2−^ (5)

Sn(OH)_6_^2^^−^ → SnO_2_↓ + 2OH^−^ + 2H_2_O (6)

**Figure 5:**
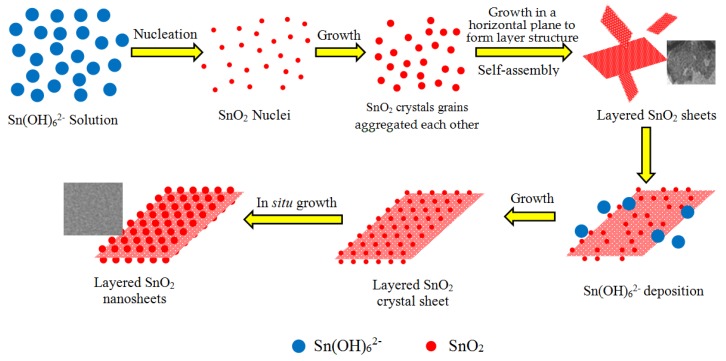
Schematic illustration of the formation mechanism of the as-prepared SnO_2_ nanosheets.

Under the hydrothermal conditions, high temperature and pressure induce the Sn(OH)_6_^2−^ ions to decompose into the SnO_2_ nanocrystals according to Reaction (6). With the hydrothermal reaction proceeding, the initial SnO_2_ nuclei grow into crystals with different sizes. According to Ostwald ripening mechanism [22], to reduce the Gibbs free energy of the system, molecules on the surface of the crystals dissolve in the solvent, and then deposit on the larger crystal’s surface. Finally, the smaller crystal grains disappear and the larger crystal grains are formed. On the other hand, driven by the high surface energy, the initial crystal grains are aggregated side-by-side in an orderly manner to self-assemble layered SnO_2_ via an oriented attachment growth. In order to minimize the total surface area, it can be considered that the orderly attachment growth in the same horizontal plane is a priority [22], which is observed from Figure 4a. Hence, it is acceptable that these crystal grains form a layered sheet structure through the oriented attachment growth. This initial layered sheet structure is important for the formation of the later SnO_2_ nanosheet in the absence of substrates. According to Bailey’s works [23,24], the formation of the mesoscale assembly process may be based on self-organization of crystalline building blocks through the spontaneous coalescence of primary nanoparticles into colloidal aggregates. The driving force for this spontaneous oriented attachment is that the elimination of the pairs of high-energy surfaces will lead to a substantial reduction in the surface energy. Subsequently, the layered sheet structure of SnO_2_ nanocrystal grains is formed by in situ growth. The growth progress of nanoparticles relates to the intrinsic anisotropic characters of the hexagonal rutile SnO_2_ crystal structure. For the rutile SnO_2_ structure, the sequence of surface energy in different crystallographic orientations is (001) > (101) > (100) > (110). Therefore, according to the lowest energy principle, active sites of the nuclei offered may induce the nanoparticles to grow along with the most stable crystal face of (110) and (101) (natural growth faces) [25,26]. The schematic illustration of the possible formation process and growth mechanism of the SnO_2_ nanosheets is depicted in Figure 5. In this work, all hypotheses can be confirmed by HRTEM images shown in Figure 4 and the result of XRD is shown as Figure 2. Of note is that the (101) surface has not been investigated very often in other reports. 

Raman was employed to analyze the lattice defects and surface properties of the as-prepared products [27,28]. It is well known that the tetragonal rutile structure SnO_2_ has six atoms (two Sn and four O ions) in each unit cell, and the symmetry normal lattice vibrational modes at the Γ point of the Brillouin zone are given on the basis of group theory [29]:

*Γ* = *Γ*^+^_1_(*A*_1*g*_) + *Γ*^+^_2_(*A*_2*g*_) + *Γ*^+^_3_(*B*_1*g*_) + *Γ*^+^_4_(*B*_2*g*_) + 2*Γ*^−^_5_(*E_g_*) + 2*Γ*^−^_1_(*A*_2*u*_) + 2*Γ*^−^_4_(*B*_1*u*_) + 4*Γ*^+^_5_(*E_u_*) (7)

**Figure 6:**
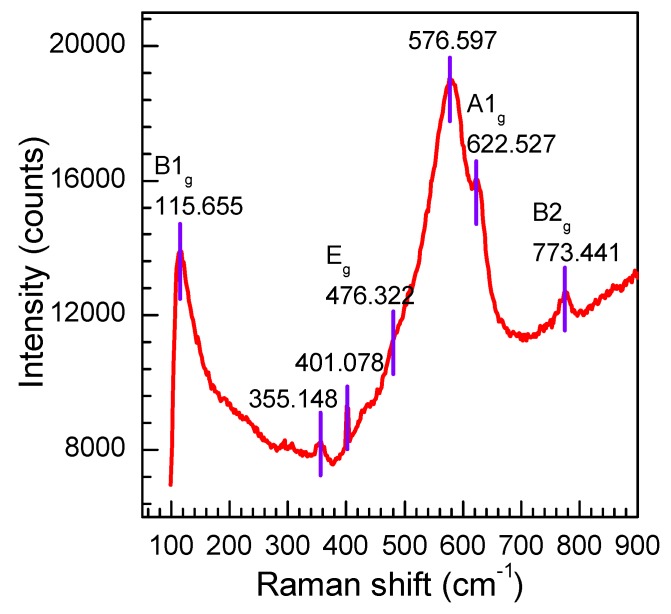
Raman scattering spectrum of the SnO_2_ nanosheets.

The Raman active modes of *B*_1*g*_, *E_g_*, *A*_1*g*_, and *B*_2*g*_ are permitted on rutile SnO_2_ with the point group of D^I4^_4h_ and the space group of P_42/mnm_ [30]. Figure 6 shows the Raman scattering spectra of the as-prepared products. The peaks located at 115.655, 476.322, 622.527, and 773.441 cm^−1^ correspond to *B*_1*g*_, *E_g_*, *A*_1*g*_, and *B*_2*g*_ vibrations, respectively, which agreed with the results reported by Lu et al. [31]. This confirms that the as-prepared product is the tetragonal rutile SnO_2_. Moreover, a broad and strong peak at 576.597 cm^−1^ and a weak peak at 355.148 cm^−1^ is observed in the spectra. These two peaks were also observed by Cheng et al. [32]. It has been proved that they are not the Raman active modes of tetragonal rutile SnO_2_ and cannot be observed in general. That they are observed here may be related to the facet surface area of the nanosheets, which are caused by the small size effect of the nanoparticles. In addition, a new peak at 401 cm^−1^ is also detected in the spectra. Although this vibrational mode needs further study, it is believed that it is attributed to the reduction of the nanoparticle size or high concentration of surface defects, such as oxygen vacancies according to the relaxation of the Raman selection rule. As a conjecture, the small size and surface defects of the SnO_2_ nanosheets may be a positive effect on the gas sensitivity of the sensor. 

**Figure 7:**
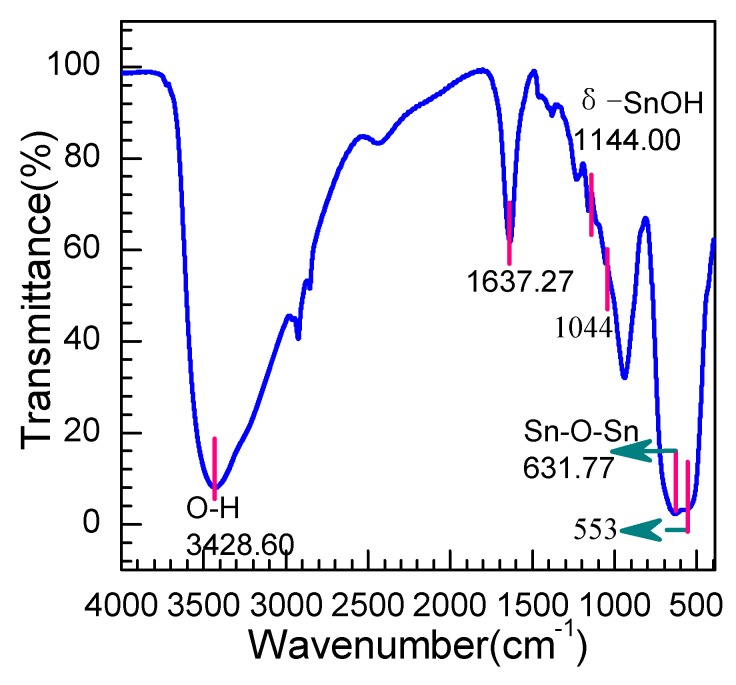
FT-IR spectrum of the as-prepared SnO_2_ nanosheets.

From the IR spectra shown in Figure 7, several absorption peaks are observed at wavelengths of 3428, 1637, 670, and 553 cm^−1^, respectively. The peaks around 3428.60 and 1637.27 cm^−1^ are ascribed to hydroxyl group stretching vibrations and bending mode vibrations associated with the absorption of a few molecules of water [33], which suggests a good adsorption capability of the as-prepared products. The bonds in the wavelength range from 1500 to 1060 cm^−1^ are ascribed to a bending mode of different types of trace hydroxyl hybrid produced of the as-prepared SnO_2_, such as the weak peak at 1144 cm^−1^ being due to the δ–(SnOH) stretching vibrations [34]. The key characteristic peaks at low wavenumbers, including 632 cm^−1^ and 553 cm^−1^, are assigned to the antisymmetric and symmetric Sn–O–Sn vibration [35], which is derived from the active IR modes E_u_ (TO) mode at 618 cm^−1^ and the A_2u_ (TO) mode at 477 cm^−1^ [36]. The shift of peaks toward the higher wavenumber may be related to the small nanoparticle size and the morphology of the SnO_2_ nanosheets.

**Figure 8:**
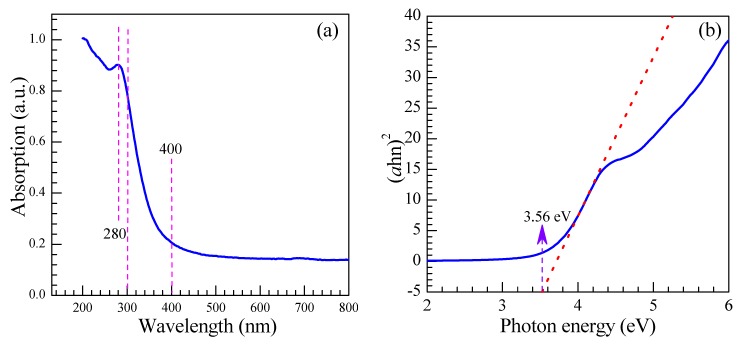
(**a**) UV-VIS diffuse reflectance spectrum of as-prepared SnO_2_ nanosheets and (**b**) (αh*v*)^2^ – h*v* photon energy curve.

The UV-VIS spectrum of the as-prepared SnO_2_ nanosheets in the wavelength range of 200 to 800 nm is shown in Figure 8a. It indicates that a strong absorption has taken place in the ultraviolet region near the visible light. However, beyond that, a weak absorption peak around 275 nm can be seen in the spectrum, which is also observed by Luo et al. [37]. As displayed in Figure 8b, the optical band gap (*E_g_*) of the as-prepared SnO_2_ nanosheets is calculated to be 3.56 eV via the equation: (αh*v*)^2^ = *B*(h*v − E_g_*). It is found to be close to 3.6 eV, which is the theoretical value of the tetragonal rutile SnO_2_. 

**Figure 9:**
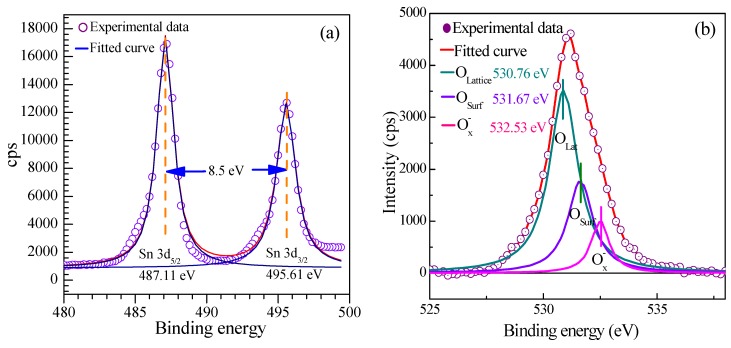
High-resolution XPS spectra of as-prepared SnO_2_ nanosheets. (**a**) Sn 3d and (**b**) O 1s.

The XPS spectra were carried out to survey the chemical composition and the surface valence state of the as-prepared SnO_2_ nanosheets. The measured spectra was decomposed into Gaussian components with a Shirley background using XPSPEAK. Figure 9 shows the Sn 3d and O 1s spectra of SnO_2_ nanosheets, respectively. Figure 9a depicts the better symmetrical high-resolution XPS spectra of Sn 3d. The classical doublet 3d_3/2_–3d_5/2_ splitting peaks at 495.61 and 487.11 eV can be assigned to the lattice tin in SnO_2_. The 8.50 eV of the energy difference between Sn 3d_5/2_ and Sn 3d_3/2_ is in good agreement with the energy splitting reported for SnO_2_ [11]. The Sn 3d_5/2_ peak is closely consistent with the standard data of SnO_2_ (486.70 eV), indicating that Sn is present in a chemical state of +4. As shown in Figure 9b, the narrow scan XPS spectrum of the O 1s demonstrates the broad and asymmetric characteristics, suggesting various coordinations of oxygen in the nanosheets. The obtained O1s peak can be carefully convoluted into three symmetrical peaks located at 530.76 eV, 531.67 eV, and 532.53 eV, respectively, indicating the presence of three kinds of oxygen species in the nanosheets. The main peak centered at 530.23 eV is assigned to oxygen ions in the SnO_2_ crystal lattice (O lattice) under completely-oxidized stoichiometric conditions [38]. The peaks located at 531.67 eV can be ascribed to the defective oxygen caused by oxygen vacancy (V_O_), oxygen interstitial (O_i_), and oxygen antisite (O_Sn_) [39], suggesting a high hole concentration within oxygen-deficient region. Those holes can promote the absorption of oxygen from the atmosphere. The peak located at 532.53 eV stems from the absorbed O_x_^−^ species (O^−^ or O^2−^ ions), which indicates that rich oxygen species adsorbed on the surface of the SnO_2_ nanosheets. The adsorbed O_x_^−^ ions are reactable with the ethylene glycol gas molecules, which then enhance the holes concentration. Thus, the increase of adsorbed oxygen ions can promote the gas sensitivity of the SnO_2_ nanosheets.

**Figure 10:**
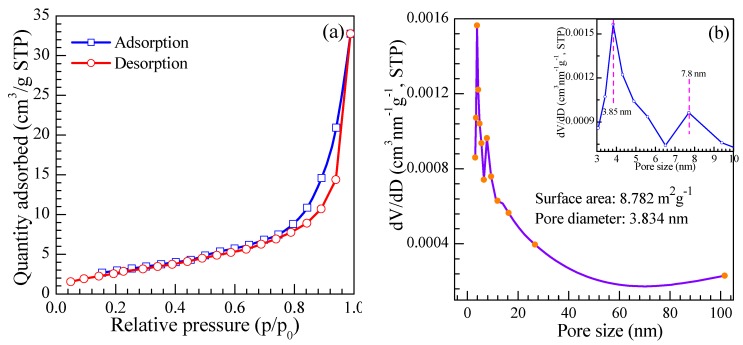
(**a**) Typical nitrogen adsorption-desorption isotherm; (**b**) BJH pore size distribution plots of as-prepared SnO_2_ nanosheets. The inset is the enlarged pore-size distribution calculated by the BJH approach from the desorption branch of the SnO_2_ nanosheets.

Figure 10a exhibits the typical nitrogen adsorption-desorption isotherm of the as-prepared nanosheets. The isotherm of the SnO_2_ products displays a hysteresis loop at relative pressure ranging from 0.05 to 0.95, being attributed to the injection and release of N_2_ in the as-prepared SnO_2_ caused by capillary condensation. This illustrates the existence of mesopores between the SnO_2_ nanoparticles. Meanwhile, the capillary condensation step is sharp, which indicates a narrow distribution of pore size. From the pore size distribution plot of the SnO_2_ nanosheets calculated by the BJH method, as shown in Figure 10b, a narrow peak is observed in the pore size ranging from 0–10 nm. The inset image shown in Figure 10b depicts that the top value of pore size is located at 3.834 nm. These pores will offer more space for adsorption of the gas to stay, so that the diffusion and reaction of gas molecules can be carried out better. The specific surface area of the as-prepared product is calculated to be 68.782 m^2^/g by the BET method. The relatively large specific surface may be advantageous for the chemical adsorption of gas molecules during the gas sensing process.

### 3.2. Gas Sensing Performance and Gas-Sensing Mechanism

The large specific surface area and thin thickness of the nanosheets render them very useful candidates for the gas sensing material with unique response properties. This is due to the fact that all the component SnO_2_ nanoparticles in the subnanometer-thick 2D nanosheet are exposed to its surface and can remarkably interact with the foreign gas species.

**Figure 11:**
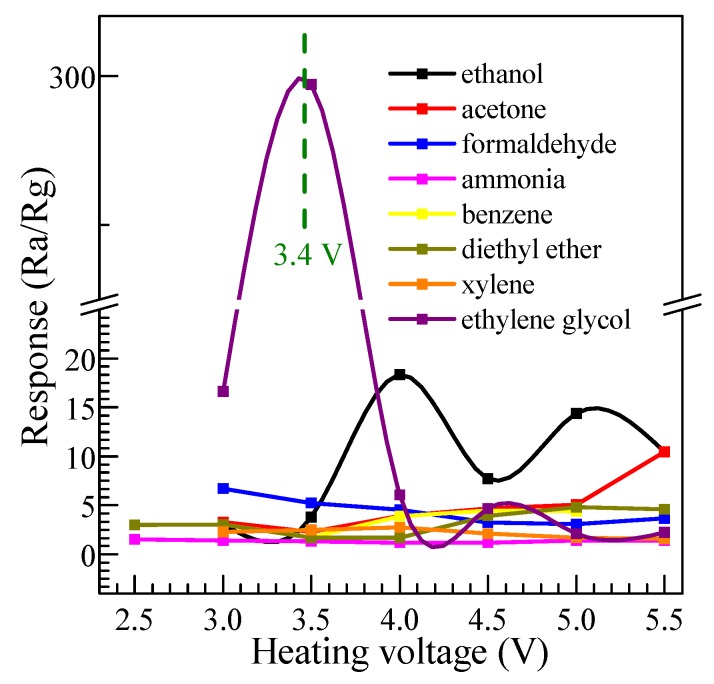
The response of the sensor to different heating voltages at 300 ppm.

In order to investigate the influence of operating voltage and response to different gases, a series of tests were performed for various gases to the concentration of 300 ppm at an operating voltage ranging from 2.5 to 5.5 V, and the results are shown in Figure 11. It demonstrates a similar change trend of the response to all kinds of gases. With the increase of operating voltage, the response increases firstly, reaches its maximum at a suitable temperature, and then decreases rapidly. This phenomenon can be explained by the mechanism of oxygen adsorption and desorption on the surface of SnO_2_ [40]. As an n-type metal oxide semiconductor, SnO_2_ can adsorb oxygen from the air in the species of O_2_^−^ and O^−^ at different temperatures. Among of them, the O^−^ can react violently with reducing volatile organic compound vapors, such as ethylene glycol, while the interaction of the O_2_^−^ between the volatile organic compound vapors is slower, comparatively. Hence, the operating temperature has a significant influence on the response for volatile organic compound vapors. At a relatively low heating voltage (operating temperature), the sensor shows a lower response because the dominant process, in this case, is the superficial adsorption of O_2_^−^. With the increase of the operating voltage, the dominant process becomes the O^−^ adsorption and the response increases, whereas, if the heating voltage increases continuously, the process turns into the adsorption of all oxygen ionic species and the response decreases [41]. From Figure 11, it can be found that the sensor made of SnO_2_ nanosheets exhibits an excellent response to ethylene glycol. The maximum of the gas sensitivity achieves 295 at the lower heating voltage 3.4 V. However, the maximum response to ethanol, acetone, formaldehyde, ammonia, benzene, diethyl ether, and xylene, at their respective optimum heating voltages, is much lower. It can be calculated that the response to ethylene glycol is 16, 28, 44, 199, 66, 62, and 110 times higher than that of those gases. Even for ethanol, the maximum response reaches 18 times higher at the optimum heating voltage of 4.0 V. Obviously, the gas sensor based on SnO_2_ nanosheets shows the high response, good selectivity, and lower optimum temperature for ethylene glycol. As a surface-controlled n-type semiconductor, the gas-sensing properties of SnO_2_ are dominantly controlled by surface adsorption, which made the surface energy and conductance change. In other words, the selectivity of the sensor is affected by several factors, such as LUMO (lowest unoccupied molecule orbit) energy of the gas molecule, activation energy of the reaction between gas molecules, and adsorbed oxygen and the amount of gas adsorption on the sensing material at different operating temperatures. For a different value of the LUMO energy for the tested gases, the energy needed for the gas-sensing reaction will be different. This indicates that the needed conditions of the reaction between the gas molecules and the adsorbed oxygen is not the same for different gases. Thus, the sensors can detect the different target gases at suitable operating temperatures. The SnO_2_ nanosheet material here shows a comparably lower response to ethanol, acetone, formaldehyde, ammonia, benzene, diethyl ether, and xylene, which means relatively good selectivity. 

**Figure 12:**
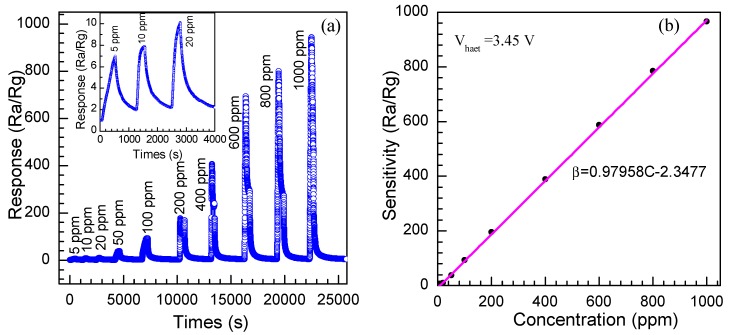
(**a**) Dynamic response-recovery periods curve of the sensor to ethylene glycol gas with the concentration ranging from 5 to 1000 ppm at the optimum operating voltage of 3.4 V. Inset: the enlarged response-recovery periods curve ranging from 5 to 20 ppm; (**b**) Linear relationship between the gas response and different concentrations of ethylene glycol.

The dynamic response and recovery process of the SnO_2_ nanosheet sensor were investigated and the results are shown in Figure 12a. Ten cycles are successively recorded at the optimum operating voltage of 3.4 V, corresponding to concentrations of 5, 10, 20, 50, 100, 200, 400, 600, 800, and 1000 ppm, respectively. As performed in Figure 12a, the curve rises fast when the ethylene glycol is injected into the test system and declines rapidly when the ethylene glycol is released. The response amplitude of the sensors gradually increases with the increasing gas concentration from 5 to 1000 ppm. The response and recovery characteristics are almost reproducible, which indicates the stability of the response of the SnO_2_ nanosheets. All of those are important characteristics for the commercial use of any electronic devices. Noticeably, as shown in the inset of Figure 12a, the sensor presents a considerable response of 6.9 to low concentrations of 5 ppm, 7.8 to 10 ppm and 12.0 to 20 ppm, suggesting the sensor based on SnO_2_ nanosheets is favorable to detect ethylene glycol with lower concentration.

The correlation curve between concentration of the ethylene glycol and the response function of the SnO_2_ nanosheet sensor at the optimum heating voltage of 3.4 V is depicted in Figure 12b. It can be seen that the response of the sensors increased with the increase of gas concentration. The responses of the nanosheets sensor reaches 38.1, 92.9, 175.5, 298.7, 682.5, 785.4, and 936.7 at 50, 100, 200, 400, 600, 800, and 1000 ppm, respectively. A linear dependence of the sensitivity on the ethylene glycol concentration is observed from 5 to 1000 ppm, suggesting that our sensor material will be able to measure a wide concentration range in practical application. It can be considered that the linear dependence of the response on concentration may be associated with the effect of the small size. The straight line of the response to the concentration of ethylene glycol is the calibration curve and the experimental data are fitted as:

*R* = 0.97958[*C*] − 1.3477 (8)

where [*C*] is the concentration of ethylene glycol gas and *R* is the response. According to the results, a lower detection limit of 1.3758 ppm can be extrapolated to ethylene glycol in dry air. Generally, the gas response of the semiconductor metal-oxide gas-sensitive resistors can be empirically represented as [42]:

*R* = *A*[*C*]*^β^* (9)

where *A* is an empirical parameter, [*C*] is the concentration of the target gas, and exponent *β* has some rational fraction value (the value is usually 1 or ½, depending on the size of the particles). Ogawa et al. suggested that *β* could be assumed to be 1 for SnO_2_ with a diameter around 15 nm, which illustrated a linear relation between the response and concentration, and 0.5 for SnO_2_ nanoparticles with a diameter larger than 20 nm [43]. Moreover, such similar results of linear dependence were also observed in 4–15 nm thin SnO_2_ nanorods [44]. Therefore, the above reports all indicate that the linear dependence is related to the small size effect of SnO_2_ materials. However, the detailed reason needs further investigation since the sensing property of SnO_2_ nanomaterials is not only affected by the small size effect, but also related to their exposed crystalline surface [45,46].

**Figure 13:**
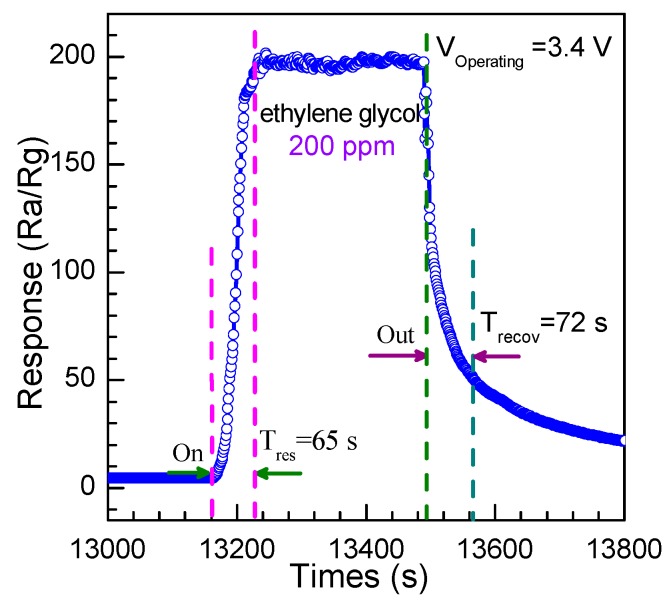
The response and recovery time of the gas sensor to 200 ppm ethylene glycol at the optimum operation voltage.

The time of the response and recovery are key parameters to evaluate the performance of the gas sensor. The response time is defined as the time when the resistance becomes stable after the injection of ethylene glycol, while the recovery time is defined as the time when the resistance returns to 90% of the original value in the air. The response curve of the sensor to 200 ppm ethylene glycol gas at 3.4 V is displayed as Figure 13. One could obvserve that the response time is 65 s and the recovery time is 72 s, suggesting a relatively slightly longer time of response and recovery. This may be ascribed to the slower speed of the adsorption and desorption of ethylene glycol, and the vast pores with sizes of about 3.834 nm between each nanoparticles and the nanosheets, which acted as reaction rooms to force oxygen and ethanol molecules to stay long enough to complete the gas-sensing reaction.

**Figure 14:**
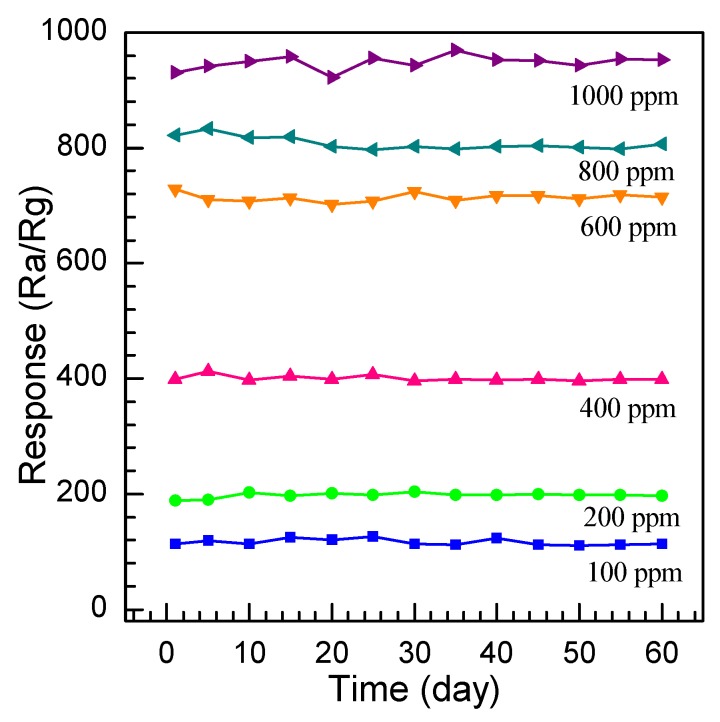
The stability of the SnO_2_ nanosheet sensor.

Finally, the long-term stability of the nanosheet gas sensors is inspected. The gas response evolution of the SnO_2_ nanosheet sensors was measured by repeating the measurement a number of times at concentrations of 100, 200, 400, 600, 800, and 1000 ppm at the optimum operation voltage of 3.4 V for 60 days. Figure 14 demonstrates the response changes are measured on the 5th, 10th, 15th, 20th, 25th, 30th, 35th, 40th, 45th, 50th, 55th, and 60th day after the first measurement. It can be calculated that the slight vibration of the 60-days-later response is ±8.3%, ±5.8%, ±6.5%, ±10.2%, ±9.4%, and 11.2% for 100, 200, 400, 600, 800, and 1000 ppm ethylene glycol gas, respectively, which illustrates a good stability and reliability of the SnO_2_ nanosheet sensor for practical application.

Until now, gas sensors for glycol detection have been rarely reported. Recently, there exist a few reports on ethylene glycol sensors that are based on porous SnO_2_ nanotubes and SnO_2_ nanosheets [47,48,49]. A comparison between the sensing performance of the SnO_2_ nanosheets and these SnO_2_ nanostructures previously reported is summarized in Table 1. As can be seen, the sensor made of SnO_2_ nanosheets in the present work displays a higher response than those reported in other studies, which confirms that SnO_2_ nanosheets have an obvious advantage over the others for ethylene glycol gas sensing. This indicates that the as-prepared SnO_2_ nanosheets could be a competitive candidate for ethylene glycol gas-sensing detection.

### 3.3. Gas Sensing Mechanism

**Figure 15:**
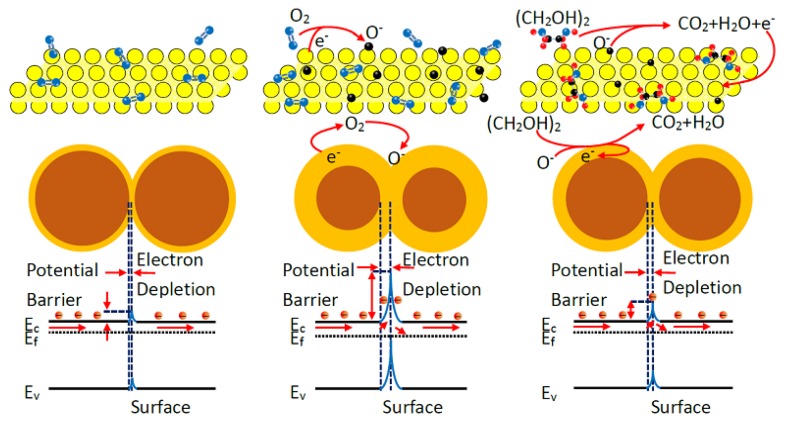
The schematic illustration of the sensing mechanism of SnO_2_ nanosheets to ethylene glycol.

The gas sensing performance of the surface-controlled n-type semiconductor can be ascribed to the change of electrical conductivity caused by the chemical interaction between gas molecules and the SnO_2_ nanosheet surface. When the sensor is exposed in the air, the oxygen molecules adsorbed on the SnO_2_ nanosheets can capture electrons from the surface and transfer oxygen into oxygen ions such as O_2_^−^, O^−^, and O^2^^−^ (Reaction (10)) [8]. These oxygen ions result in the formation of an electron, depleted layer within the SnO_2_ surface, which can lead to the decline of the Fermi level and a large increase of the potential barrier. As a consequence, the electrical conductivity will reduce and the electrical resistance will increase, obviously. When the target gas, such as ethylene glycol, is injected, the gas molecules are oxidized by O^−^ and O^2^^−^ adsorbed on the surface (Reaction (10)). Those electrons captured by O_2_ molecules are released to the conduction band of SnO_2_, which cause the depletion layer thickness decrease. At the same time, the Fermi level shifts upwards and the potential barrier reduces, resulting in the electrical resistance decrease. The detailed gas sensing process and mechanism is displayed in Figure 15 and the whole reaction process can be illustrated by Reactions (10) and (11):

O_2(gas)_ → O_2(ads)_ +e^−^ → O_2_^−^_(ads)_ + e^−^ → 2O^−^_(ads)_ + 2e^−^ → 2O^2−^_(ads)_ (10)

(CH_2_OH)_2(gas)_ → (CH_2_OH)_2(ads)_ + O^2^^−^_(ads)_ → CO_2_ + H_2_O + e^−^ (11)

Based on the above gas-sensing results, it shows that the sensor fabricated by the SnO_2_ nanosheets exhibits an excellent response to ethylene glycol at a lower operating temperature. A possible mechanism of the excellent gas-sensing properties of SnO_2_ nanosheets is discussed here. The excellent gas-sensing properties of nanosheets is the result of the synergistic effect of multiple factors. On the one hand, the excellent ethylene glycol response is attributed to the large and continuous area and smooth surface of the nanosheets. The large specific surface area can enrich absorbed oxygen on the surface of the SnO_2_ nanosheets, which is confirmed by the XPS results, as shown in Figure 9b. It can provide more active sites for gas adsorption on the surface, contributing to a higher sensor sensitivity. On the other hand, the grain size (*D*) of SnO_2_ nanosheets has a great influence to the gas sensing performance of the sensors. As discussed above, when SnO_2_ nanosheets are exposed to air, the chemisorption of oxygen leads to the formation of a depletion region within the surface. Upon exposure to ethylene glycol vapor, the trapped electrons are released back into the conduction band of SnO_2_ and the thickness of the depletion region decreases, resulting in the sensor resistance decreasing. When a crystallite size is comparable to the Debye screening length (*D*~2*L*_D_), the depletion layer has a strong effect and the sensor sensitivity is strongly dependent on an average crystallite size. It was reported that the value *L*_D_ is about 7 nm for SnO_2_ [50]. Therefore, the crystal grains with a size smaller than 14 nm (2*L*_D_) will contribute more to the enhancement of SnO_2_ electrical conductivity upon ethylene glycol vapor exposure. Such consideration resolves around the observed response results as displayed in Figure 11, where the SnO_2_ nanosheet-based sensor shows a higher sensitivity to ethylene glycol vapor due to the structural evidence that SnO_2_ nanosheets are composed of crystallites (grains) with an average size smaller than 2*L*_D_. Moreover, the structural result shows that crystal grains of SnO_2_ nanosheets expose the dominant facet of (101), which is the high-energy facet compared with the (110) facet of the SnO_2_. As is well known, the high energy of the exposed facets, such as (211) or (111), always lead to an active surface due to high-density atom steps, ledges, and dangling bonds [51]. Therefore, those high energy of the exposed facets of (101) will enhance the activity of the gas molecules chemisorption, turning to prompt the response of the ethylene glycol vapor. Although, it is involuted that the reasons of the SnO_2_ nanosheets exhibit excellent response to ethylene glycol and needs to be further studied in detail, the mechanism can be explained by the aspects discussed above.

## 4. Conclusions

In summary, SnO_2_ nanosheets assembled by nanoparticles with diameters of 6–12 nm have been successfully synthesized by a one-step facile hydrothermal method without substrates or surfactants at a lower temperature of 130 °C. The microstructure of the SnO_2_ nanosheet evidences a tetragonal rutile structure with a band gap value of 3.56 eV. The dominant exposed surface of the nanoparticles of the SnO_2_ nanosheets is (101), but not (110). As the gas-sensing materials, the as-prepared SnO_2_ nanosheet exhibits an excellent sensing response toward ethylene glycol at a lower operating voltage of 3.4 V. The response to 400 and 1000 ppm ethylene glycol reaches 395 and 937 at 3.4 V, respectively. We believe that the larger specific surface, the small size of the nanoparticles with a diameter close to the Debye length, and the high-energy exposed facets of (101) with a large number of effective active sites are the main possible reasons for the excellent gas performance toward ethylene glycol. This work suggested that the SnO_2_ nanosheets could be efficiently applied as the gas-sensing material to detect ethylene glycol. The excellent sensing response will excite the SnO_2_ nanosheets to be a competitive candidate for ethylene glycol gas-sensing detection.

## Figures and Tables

**Table 1:** Comparison of the sensing properties of SnO_2_ nanosheets and other reported SnO_2_ nanostructures.

Sensing Materials	Fabrication Approach	Average Grain Size (nm)	Glycol Concentration (ppm)	Sensor Response (*R_a_*/*R_g_*)	Heating Voltage (V)/Temperature (°C)
Porous SnO_2_ nanotubes [47]	Hydrothermal route using template	11.3	20	17.2	300
Zn-doped SnO_2_ hierarchical nanostructures [48]	One-step hydrothermal route	23	100	90	240
Hierarchical Zn-doped SnO_2_ nanosheets [49]	Hydrothermal route	40	100	43	240
SnO_2_ nanosheet (Present work)	Hydrothermal route	6–12	300/1000	295/937	3.4/~220
